# Neutrophil extracellular traps and citrullinated fibrinogen contribute to injury in a porcine model of limb ischemia and reperfusion

**DOI:** 10.3389/fimmu.2024.1436926

**Published:** 2024-09-09

**Authors:** Valentina Zollet, Isabel Arenas Hoyos, Stefanie Hirsiger, Bilal Ben Brahim, Maria Francesca Petrucci, Daniela Casoni, Junhua Wang, Rolf Spirig, Kay Nettelbeck, Luisana Garcia, Lena Fuest, Esther Vögelin, Mihai Constantinescu, Robert Rieben

**Affiliations:** ^1^ Department for BioMedical Research (DBMR), University of Bern, Bern, Switzerland; ^2^ Graduate School for Cellular and Biomedical Sciences (GCB), University of Bern, Bern, Switzerland; ^3^ Department of Plastic and Hand Surgery, Inselspital, University Hospital Bern, Bern, Switzerland; ^4^ Experimental Surgery Facility, Experimental Animal Center, University of Bern, Bern, Switzerland; ^5^ Commonwealth Serum Laboratories (CSL) Behring, Research, Commonwealth Serum Laboratories (CSL) Behring Biologics Research Center, Bern, Switzerland

**Keywords:** ischemia/reperfusion injury (IRI), large animal model, porcine model of limb IRI, neutrophil extracellular traps (NETs), citrullination, citrullinated plasma proteins, coagulation, citrullinated fibrinogen

## Abstract

**Background:**

Ischemia/reperfusion injury (IRI) is a complex pathological process, triggered by the restoration of blood flow following an interrupted blood supply. While restoring the blood flow is the only option to salvage the ischemic tissue, reperfusion after a prolonged period of ischemia initiates IRI, triggering a cascade of inflammatory responses ultimately leading to neutrophil recruitment to the inflamed tissue, where they release neutrophil extracellular traps (NETs). NETs are web-like structures of decondensed chromatin and neutrophilic proteins, including peptidyl-arginine deiminase 2 and 4 (PAD2, PAD4), that, once outside, can citrullinate plasma proteins, irreversibly changing their conformation and potentially their function. While the involvement of NETs in IRI is known mainly from rodent models, we aimed to determine the effect of NET formation and especially PADs-mediated extracellular protein citrullination in a porcine model of limb IRI.

**Methods:**

We conducted our study on amputated pig forelimbs exposed to 1 h or 9 h of ischemia and then reperfused *in vivo* for 12 h. Limb weight, edema formation, compartmental pressure were measured, and skeletal muscle was analyzed by immunofluorescence (TUNEL assay and dystrophin staining) to evaluate tissue damage. Fibrin tissue deposition, complement deposition and NETs were investigated by immunofluorescence. Citrullinated plasma proteins were immunoprecipitated and citrullinated fibrinogen was identified in the plasma by Western blot and in the tissue by immunofluorescence and Western blot.

**Results:**

Our data consolidate the involvement of NETs in a porcine model of limb IRI, correlating their contribution to damage extension with the duration of the ischemic time. We found a massive infiltration of NETs in the group subjected to 9 h ischemia compared to the 1 h and citrullinated fibrinogen levels, in plasma and tissue, were higher in 9 h ischemia group. We propose fibrinogen citrullination as one of the mechanisms contributing to the worsening of IRI. NETs and protein citrullination represent a potential therapeutic target, but approaches are still a matter of debate. Here we introduce the idea of therapeutic approaches against citrullination to specifically inhibit PADs extracellularly, avoiding the downstream effects of hypercitrullination and keeping PADs’ and NETs’ intracellular regulatory functions.

## 1 Introduction

Ischemia/reperfusion injury (IRI) is a pathological inflammatory process that occurs when a tissue experiences reperfusion after a period of interrupted blood supply. It is a condition observed in several clinical scenarios, including stroke, myocardial infarction, thrombosis, transplantation, or prolonged tourniquet application on an extremity due to severe trauma (1).

The tolerance of tissues for ischemia varies with the nature of the tissue, the presence or absence of collateral flow, temperature, and tissue mass. In general, muscle is highly susceptible to ischemia, with a tolerance of up to 4 h, followed by nerves, with reversible ischemic changes up to 8 h, fat up to 13 h, skin up to 24 h, and bone up to four days at normothermia (2). It is known that in a limb, the skeletal muscle, which represents the majority of tissue in an extremity, is most vulnerable to ischemia (2–4).

Tissue injury and/or death occur as a result of the initial ischemic insult, which is determined primarily by the magnitude and duration of the interruption in the blood supply, and then subsequent damage induced by reperfusion (2). During prolonged ischemia, ATP levels and intracellular pH decrease as a result of anaerobic metabolism and lactate accumulation. As a consequence, ATPase-dependent ion transport mechanisms become dysfunctional, contributing to increased intracellular and mitochondrial calcium levels (calcium overload) (5, 6), reactive oxygen species (ROS) production (7), cell swelling and rupture, and ultimately cell death. Subsequently, damage-associated molecular patterns (DAMPs), cytokines and chemokines are released, adhesion molecules are upregulated, endothelial cell dysfunction occurs (8), glycocalyx shedding takes place (9), and complement (10, 11) and coagulation pathways are activated. This cascade of events worsens the initial damage caused by ischemia, fueling a pro-inflammatory feedback loop that leads to IRI (12, 13).

These inflammatory events lead to complement activation, resulting in the cleavage of C3 and C5, which generate the anaphylatoxins C3a and C5a. This cascade continues downstream, culminating in the formation of the membrane-attack complex (MAC; C5b-9). All of these components play pivotal roles in the amplification of the inflammatory response, chemotaxis, neutrophil recruitment and activation, and lately direct cell injury (14).

Once primed, leukocytes form loose adhesive interactions with the endothelium, followed by firm adhesion if the inflammatory stimulus is sufficient. Subsequently, leukocytes egressing from the vascular bed exacerbate ischemic injury by disrupting the microvascular barrier and increasing microvascular permeability, transcapillary fluid filtration and edema formation. After their diapedesis into the perivascular space, neutrophils contribute to cell injury through several effector functions. They are among the first cells to be recruited to the inflammatory site within 1 hour after reperfusion (4, 15). Once activated, they contribute to muscle cell damage and death through additional ROS production, release of their granules containing myeloperoxidase, defensin, gelatinase, elastase, lactoferrin and extrusion of neutrophil extracellular traps (NETs) (16).

NETs are web-like structures of decondensed chromatin decorated with neutrophilic proteins such as neutrophil elastase (NE), myeloperoxidase (MPO), citrullinated histones 3 and 4 (CitH3, CitH4) and peptidyl-arginine deiminase 2 and 4 (PAD2, PAD4), among others (17, 18). PADs, once released into the extracellular environment, are still active and can citrullinate plasma proteins like serine protease inhibitors or SERPINs (C1-INH, PAI-1, tPA), ADAMTS13 (19) and fibrinogen (20–22), thus affecting the coagulation cascade.

Citrullination is a post-translational modification catalyzed by calcium-dependent PAD enzymes. Through a deamination process, PAD enzymes convert a peptidyl-arginine into a peptidyl-citrulline, leading to a loss of positive charge, which irreversibly affects the conformation and the function of the target protein (19, 23–25).

Although NETs and serine proteases are known to participate in IRI (5, 13, 26, 27) the impact of NET formation, PADs release and extracellular citrullination has not been shown in large animal, translational models of IRI or in a clinical setting. Here, we hypothesize that NET formation and consequent extracellular citrullination lead to endothelial and muscle damage, building up a vicious cycle between NETs and the activation of the plasma cascade systems complement and coagulation, leading to a triangular relationship with irreversible inflammatory effects (28).

## 2 Material and methods

### 2.1 *In vivo* porcine surgical model of limb IRI: anesthesia and analgesia management

12 wild-type Large White (Swiss Landrace) pigs, both males and females, with a body weight range of 35-45 kg were used. The experimental setup consisted of two different groups of warm ischemia (22°C): six animals were allocated to the short ischemia time (<1h) group and the other six to the prolonged ischemia time (9h) group. After clinical examination, a combination of dexmedetomidine 20 µg/kg, ketamine 10 mg/kg and methadone 0.2 mg/kg were injected intramuscularly behind the ear. When adequate sedation was achieved, oxygen supplementation via a non-tight mask (8-10 L/min) was started. A peripheral venous catheter was placed in an auricular marginal vein, and general anesthesia was induced with ketamine (1 mg/kg) and propofol to effect (1-2 mg/kg). Infusion of Ringer’s Lactate solution was started at 5 ml/kg/h. Tracheal intubation (endotracheal tube with internal diameter 7–8 mm) was performed, and intermittent positive pressure ventilation started (tidal volume 8–12 ml/kg, respiratory rate adapted to an end-tidal CO_2_ of 40–45 mmHg, positive end-expiratory pressure 5 cm H_2_O). General anesthesia was deepened and maintained with isoflurane in O_2_/air (targeting end-tidal isoflurane 0.8–1%, minimal alveolar concentration 1.8–2%). Amoxicillin/clavulanic acid (20 mg/kg) was given intravenously as prophylactic antibiotic therapy before the first surgical incision and repeated every 12 hours. Additional antinociception was provided at least 40 minutes before the limb amputation with a paravertebral single site anesthetic injection. With the animal in lateral recumbency, transverse processes and vertebral bodies of C6 and C7 were visualized with ultrasonography and a needle connected to a nerve stimulator was inserted caudally to the transverse process of C6 and directed caudo-medially to puncture the deep cervical fascia and anaesthetize the two ventral branches emerging from C7 with 0.2 ml kg-1 ropivacaine 0.75%. Further systemic analgesia was provided if autonomic reactions were observed during surgery (mean arterial pressure or heart rate increase ≥ 20% from baseline).

Central venous and arterial catheters were placed into the external jugular and external carotid respectively. A surgical incision and dissection of the vessels was performed, and the line was placed via a Seldinger technique, followed by wound closure. Intraoperative monitoring consisted of heart rate, respiratory rate, arterial oxygen saturation, capnography, invasive blood pressure (coccygeal artery and carotid artery), esophageal temperature, inspired and expired fraction of gases (air, O_2_, CO_2_), central venous pressure and electroencephalogram (EEG) through surface electrodes. Additionally, a trans-abdominal bladder catheter (percutaneous Cystofix) was placed to monitor urine output. At the end of the amputation, a wound catheter was inserted under visual control in the proximity of the brachial plexus and ropivacaine 0.75% (0.1 ml/kg) was injected every 8 hours. 10’000 IU of heparin was administered intravenously before the first incision. Blood flow in the axillary artery was then measured, followed by complete amputation of the forelimb. The ischemia time (1h or 9h at room temperature [RT, 22°C], according to the experimental group) started immediately, once the artery was clamped and the amputated limb was weighed. The vasculature of the amputated limb was rinsed with hydroxyethyl starch solution (HAES, Voluven, Fresenius, Bad Homburg, Germany) directly before surgical replantation and *in vivo* reperfusion. For the 1h ischemic group, the reimplantation started immediately after weighing the limb, whereas for the 9h ischemia group the limbs were placed in a sterile bag and stored for 9h at RT and the soft tissue defect was temporarily covered with Epigard. For replantation, subcutaneous sutures were used to hold the limb in place in the dorsal area, and venous and arterial anastomoses were carried out. Immediately before the onset of reperfusion, a bolus of heparin (80 IU/kg) was administered and followed by additional administration of 30-60 IU/kg/h. The infusion was adjusted based on the activated clotting time (ACT). The target ACT was 2-3 times the baseline (200-300 seconds). After limb replantation, a new wound catheter was inserted and ropivacaine was injected at the same dose and intervals until the end of the experiment. Fluid therapy was continued at 3 ml/kg/h until end of the experimental procedure. An adjusted rate infusion of dexmedetomidine (2-6 µg/kg/h) was initiated 6-8 hours after the first ropivacaine injection and maintained until euthanasia. Crystalloid and colloid boluses as well as inotropes and ino-pressors (i.e., calcium gluconate, dobutamine, noradrenaline) were administered when required, targeting a carotid mean arterial pressure of at least 60 mmHg. Blood gas analyses were repeated over the anesthesia duration at pre-determined time points and when clinically indicated to monitor pulmonary exchanges, glycemia, potassium values and acid-base balance. Reperfusion for 12h immediately started, while subcutaneous sutures were completed, drainage placed, and the skin closed. Subsequently, lines for compartment pressure measurement were placed in the replanted and contralateral limbs. Evaluation perfusion of the limb was carried out through Doppler analysis throughout the reperfusion time. Animals were euthanized after completion of 12h of *in vivo* reperfusion, or in case of severe complications – essentially multiorgan failure as a systemic consequence of IRI – under general anesthesia with an overdose of intravenous pentobarbital (100 mg/kg). Flat EEG and asystole confirmed the pigs’ death.

The limb was then removed and weighed, and tissue samples collected for further analysis.

### 2.2 Assessment of edema formation

Total limb weight was determined before and after reperfusion to evaluate the gross weight increase due to fluid accumulation in the limb after ischemia followed by reperfusion (ratio of limb weight post/pre-reperfusion).

To assess edema formation in the affected muscle, 10 punch biopsies of skeletal muscle tissue from ischemic-reperfused limbs were collected at endpoint and immediately weighed (wet weight). Subsequently, the collected biopsies were dried at 80°C for 40h and weighed again (dry weight). The ratio between wet weight and dry weight (wet/dry weight ratio) was calculated as an additional indicator of edema formation.

Compartmental pressure of ischemic limbs was monitored during perfusion time, as an indication of pressure changes in the muscle compartment due to fluid accumulation in the interstitium.

### 2.3 TUNEL assay

To determine whether limb IRI results in DNA fragmentation of cell nuclei, terminal deoxynucleotidyl transferase dUTP nick end labeling (TUNEL) assay was conducted using the TACS 2 TdT- Fluor *In Situ* Apoptosis Detection Kit (R&D Systems, 4812-30-K) according to the manufacturer’s instructions. Briefly, Optimal Cutting Temperature (O.C.T.) compound-embedded, fresh frozen sections were incubated with TUNEL reaction mixture (50 μl) for 1 h at 37°C in a humidified chamber in the dark. After the labeling procedure, the slides were washed, mounted with Prolong Diamond Antifade Mountant with DAPI (Invitrogen P36962) and imaged using a 20x objective on a Zeiss LSM980 confocal microscope. The percentage of TUNEL-positive nuclei/area was quantified in 10 non-overlapping fields, using Image J software (version 2.3.0/1.53q).

### 2.4 Immunofluorescence staining

Skeletal muscle tissue samples were embedded for cutting in transversal orientation in TissueTek O.C.T. compound (Sakura 4583). 6μm thick sections were fixed and permeabilized with ice-cold 1:1 acetone (AppliChem 141007.1211)/methanol (Merck 1.06009.2500) for 10 min at RT and rehydrated in TBS for 5 min. After blocking for one hour at RT with TBS-3% BSA (Merck A7030), cryosections were incubated overnight at 4°C with the following antibodies: anti-human dystrophin (Abcam ab15277, cross-reactive with the respective porcine protein), anti-human myeloperoxidase (Abcam ab25989, clone 2C7, cross-reactive with the respective porcine protein) and anti-citrullinated histone 4 (Merck 07-596, cross-reactive with the respective porcine protein) or anti-citrullinated histone 3 (Abcam ab5103, cross-reactive with the respective porcine protein), anti-porcine CD31 (R&D MAB33871) and anti-porcine complement C5b (Invitrogen DIA 011-01-02, clone aE11) or polyclonal anti-human fibrinogen (DAKO F0111, cross-reactive with the respective porcine protein) or monoclonal anti-citrullinated human fibrinogen (ImmunoPrecise MQR2.101-100, clone 1F11, cross-reactive with the respective porcine protein), all diluted in TBS-1% BSA-0.05% Tween (Tween 20, AppliChem A4974,0250). Subsequently, samples were incubated for 1h at RT with secondary antibodies: goat anti-rabbit IgG AlexaFluor680 (Invitrogen A21076), donkey anti-mouse IgG AlexaFluor568 (Invitrogen A10037), donkey anti-rabbit IgG AlexaFluor488 (Invitrogen A32790), goat anti-rat IgG AlexaFluor680 (Invitrogen A21096), goat anti-human IgG AlexaFluor488 (Invitrogen A11013). All secondary antibodies were diluted in TBS-1% BSA-0.05% Tween. Slides were washed, mounted with Prolong Diamond Antifade Mountant with DAPI (Invitrogen P36962) and imaged using a 20x objective on a Zeiss LSM980 confocal microscope and analyzed with ImageJ (version 2.3.0/1.53q).

### 2.5 Immunoprecipitation of citrullinated proteins from blood plasma

Detection of citrullination was based on the binding of phenylglyoxal (PG) to citrulline (29). Biotin-PG labeling of citrullinated plasma proteins was carried out in 1.5 ml Eppendorf tubes by mixing 300 µg of plasma protein, 15 µl of 50 mM HEPES (PAN Biotech, P05-01100P), 60 µl of 20% trichloroacetic acid (Sigma, T0699-100 ml),12 µl of 200 µM biotin-PG (Cayman, REF 17450), and Milli-Q water to reach a final volume of 300 µl. Technical control samples were prepared as described above without adding the biotin-PG probe. All samples were incubated at 37°C for 1 hour. The labeling reactions were quenched by adding 60 µl of 0.5 mM L-citrulline (Sigma C7629-5G) in 50 mM HEPES and incubated again at 37°C for 30 minutes to allow for complete quenching. Subsequently, samples were incubated on ice for 45 minutes to allow protein precipitation. Precipitated proteins were cold pelleted (4°C) at 20000 ×g for 15 minutes. Precipitates were washed twice in ice-cold acetone (AppliChem 141007.1211), allowed to air-dry for 1 hour at RT. Lyophilized labeled proteins were resolubilized by adding 1 ml of 1.2% SDS-PBS and incubated in a water bath sonicator (Bandelin, Sonorex 50/60 Hz) for at least 1h, until pellets were completely resuspended. After complete resuspension, samples were transferred into 15 ml tubes containing 5 ml of PBS to dilute the SDS to 0.2%. To pull down the PG-labeled citrullinated proteins, 160 µl of streptavidin-Sepharose bead slurry (Pierce ThermoFisher, streptavidin agarose beads 20353) were washed thrice with PBS and centrifuged at 500 ×g for 5 minutes and then added to each sample and incubated at 4°C overnight with end-over-end rotation. The following day, samples were centrifuged at 500 ×g for 5 minutes and then washed once with 0.2% SDS and thrice with PBS. Protein elution was achieved by adding 50 µl of 2x Laemmli sample buffer (Bio-Rad 1610747) and 1x Nupage sample reducing agent (Invitrogen, ThermoFisher, NP0004), incubated at 90°C for 10 minutes. Beads were spun down and 45 µl samples were loaded on SDS-PAGE gel (Bio-Rad Mini-PROTEAN TGX gels 4-20% 4561094) and transferred to nitrocellulose membrane (Invitrogen, ThermoFisher, iBlot 2NC Regular Stacks, IB23001) by an iBlot2 gel transfer device (Invitrogen, ThermoFisher, IB21001) with program P0. Membranes were blocked with Intercept (PBS) blocking buffer (LI-COR, 927-70001) for 1h at RT, then incubated with primary antibody polyclonal rabbit anti-human fibrinogen (1:500 dilution, DAKO A0080) overnight at 4°C, allowing the antibody binding to the protein of interest (citrullinated fibrinogen) among all the citrullinated plasma proteins purified with the pull-down method described above. Membranes were then washed and incubated for 1h at RT with IRDye 800CW goat polyclonal anti-rabbit IgG secondary antibody (1:5000 dilution, Licor, 926-32211) and visualized using the LI-COR Odyssey 9120 near-infrared imager.

### 2.6 Western blotting of citrullinated fibrinogen from skeletal muscle tissue

Skeletal muscle lysates (30 µg protein) were loaded on SDS-PAGE gel (Genscript SurePAGE, Bis-Tris gels 4-20%, M00656) and transferred to a polyvinylidene fluoride membrane (Invitrogen, ThermoFisher, iBlot PVDF Regular Stacks, IB24001) by iBlot2 gel transfer device (Invitrogen, ThermoFisher, IB21001) with program P0. Membranes were stained for total protein with total protein Q staining according to kit manufacturing instructions (Azure Biosystems, TotalStain Q PVDF, AC2225) and visualized with Vilber Quantum. Membranes were blocked for 30 minutes in 1% BSA, PBS, 0.05% Tween-20 and then incubated with primary antibody monoclonal human anti-citrullinated fibrinogen antibody, clone 1F11 (1:500 dilution, ImmunoPrecise MQR 2.101-100) for 2h at RT. Membranes were then washed and incubated for 1h at RT with HRP-conjugated secondary antibody polyclonal rabbit anti-human IgG (1:25000 dilution, Dako, P0214). Membranes were incubated with WesternBright ECL (Advansta K-12045-D20) and visualized on a Vilber Fusion FX device. The membranes were then further incubated for 20 minutes at RT with primary antibody monoclonal mouse anti-multispecies GAPDH, clone 1D4 (1:1000 dilution, Invitrogen, MA1-16757), washed and incubated for 20 minutes at RT with IRDye 800CW goat polyclonal anti-mouse IgM secondary antibody (1:5000 dilution, LI-COR, 926-32280) and the fluorescence signal for GAPDH (used as loading control) visualized using a LI-COR Odyssey 9120 near-infrared imager.

### 2.7 Statistical analysis

Data analysis was performed with commercially available software (GraphPad Prism 10.0.2, San Diego, CA, USA). All data reflect mean ± SD and values are shown as dots for each individual experiment. Normal distribution was tested with the Shapiro-Wilk test and outliers were identified. For comparison between two groups, unpaired Student’s *t*-test, one or two-tailed, Mann–Whitney-*U* test was used. Ordinary one-way ANOVA was run for comparison between three groups, multiple paired t-test was employed to compare matched data. Significant differences are indicated and the following symbols represent the statistical significance based on *p* values determined by the specific group tests described in each figure legend: *P<0.05, **P< 0.01, ***P<0.001 and ****P<0.0001.

## 3 Results

### 3.1 Prolonged ischemia followed by reperfusion leads to severe skeletal muscle damage

To determine the extent of muscle damage after 1h and 9h of ischemia, respectively, followed by 12h of *in vivo* reperfusion, we analyzed the weight increase of the limb, edema formation, compartmental pressure, DNA fragmentation in myocytes, and muscle tissue integrity.

**Figure 1:**
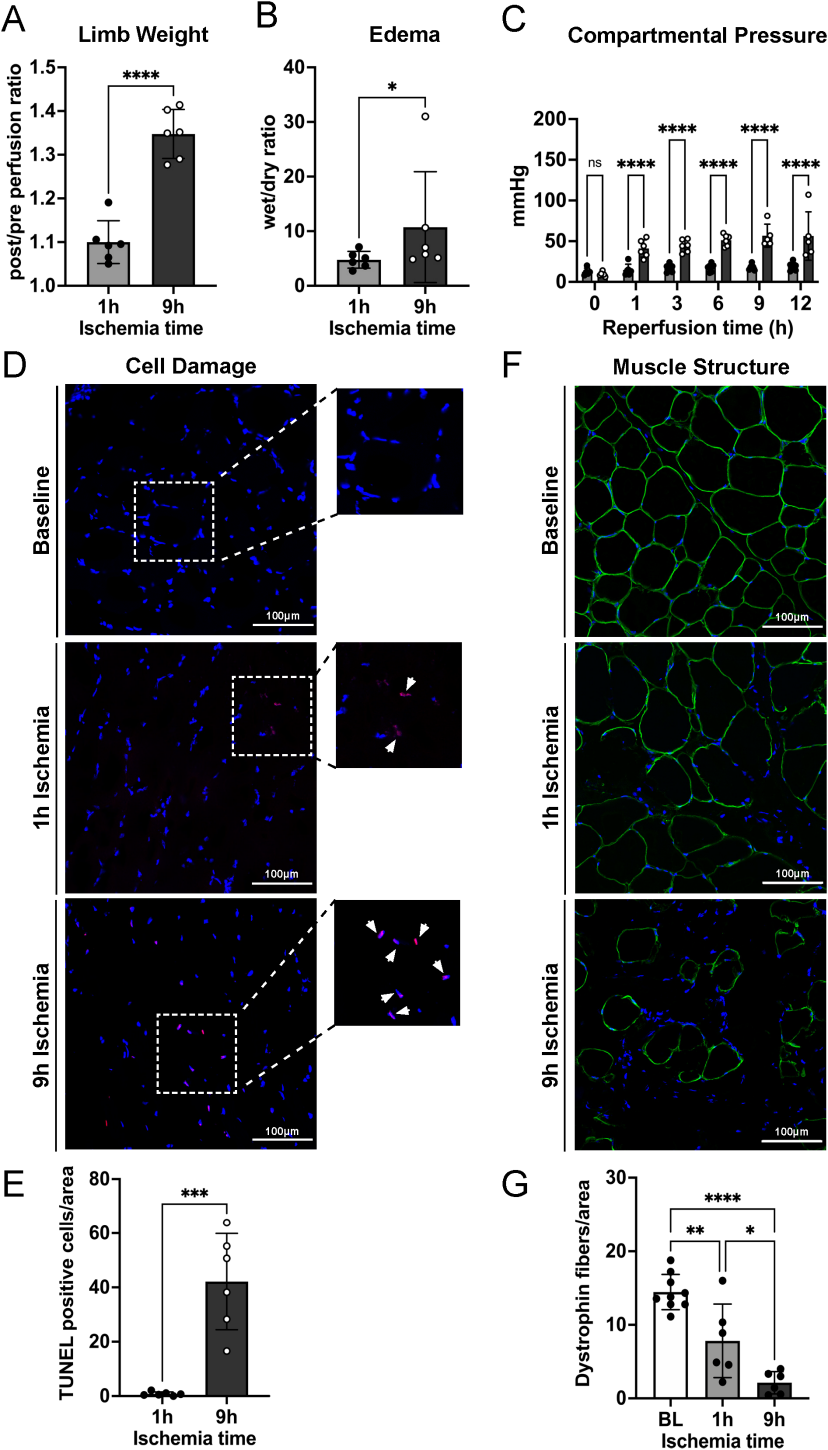
Prolonged ischemia followed by reperfusion leads to severe edema formation, DNA fragmentation in myocyte nuclei, and skeletal muscle tissue damage. **(A)** Limb weights post/pre perfusion, after 1 hour or 9 hours of ischemia and 12 hours of reperfusion were recorded, n=6/group, unpaired t-test ****P<0.0001. **(B)** Ten punch biopsies of ischemic reperfused skeletal muscle tissues were collected per limb and their wet and dry weight measured. The wet/dry weight ratio was calculated as indicator of edema formation, n=6/group, unpaired t-test, Mann-Whitney test, *P<0.05. **(C)** Compartmental pressure observed during reperfusion of limbs after 1h and 9h of ischemia, n=6/group, two-way ANOVA, mixed model ****P<0.0001. **(D)** Representative TUNEL staining images of skeletal muscle, scale bar = 100µm, n=6/group. **(E)** The percentage of TUNEL positive cells/area in 10 non-overlapping fields/animal was quantified using Image J software, n=6/group, unpaired t-test ***P<0.001. **(F)** Representative skeletal muscle tissue structure images before ischemia/reperfusion (Baseline, BL) and after 1h and 9h ischemia followed by 12h reperfusion (Endpoint). DAPI (blue), dystrophin (green), scale bar = 100µm. **(G)** Numbers of intact myofibers, dystrophin positive, in 3 non-overlapping fields/animal were quantified using Image J software, n=6/group, ordinary one-way ANOVA, Bonferroni test, *P<0.05, **P< 0.01, ****P<0.0001. Values are shown as dots for each individual experiment with indication of mean ± SD.

As expected, measurement of the limb weight before and after reperfusion revealed significantly more weight increase post-reperfusion in the 9h than in the 1h ischemia group (**Figure 1A**) associated with increased edema formation in muscle tissue assessed by the wet/dry weight ratio of muscle biopsies (**Figure 1B**). Fluid leakage from the vasculature and its accumulation in the muscle tissue leads to increased pressure into the affected compartment. Compartment pressure was therefore measured repeatedly during the 12h reperfusion and showed a significant increase already after 1h in the 9h ischemia limbs, but not in the 1h ischemia controls (**Figure 1C**). To confirm whether IRI could induce myocyte damage, DNA fragmentation was assessed by TUNEL assay. The results revealed that skeletal muscle tissue subjected to 9h ischemia followed by 12h reperfusion displayed a high number of nuclei positive for the fluorescein-dUTP labeling, indicating extensive DNA fragmentation. This was significantly higher than the TUNEL-positive nuclei observed in the 1h ischemia group (**Figures 1D, E**). Furthermore, we confirmed the extent of cellular damage in the skeletal muscle structure by means of dystrophin staining, a structural protein of the myofibers. In contrast to the well-organized fibers observed at baseline, prior to the ischemia-reperfusion injury, the cross-sectional views of the muscles exposed to 1h or 9h of ischemia displayed significant architectural distortions with increased gaps between the myofibers and a notable amount of muscle fibers almost completely destroyed. Notably, the structural damage was significantly more severe in the 9h ischemia group as compared to the 1h ischemia control (**Figures 1F, G**).

### 3.2 Prolonged ischemia followed by reperfusion leads to increased fibrin deposition in tissue

Fibrinogen plays a pivotal role in acute inflammatory processes and its intra- and extra-vascular deposition represents a widely accepted feature of tissue injury. Fibrinogen can influence several aspects of inflammatory cell function by engaging leukocytes through multiple mechanisms and misbalancing the equilibrium between coagulation and fibrinolysis.

**Figure 2:**
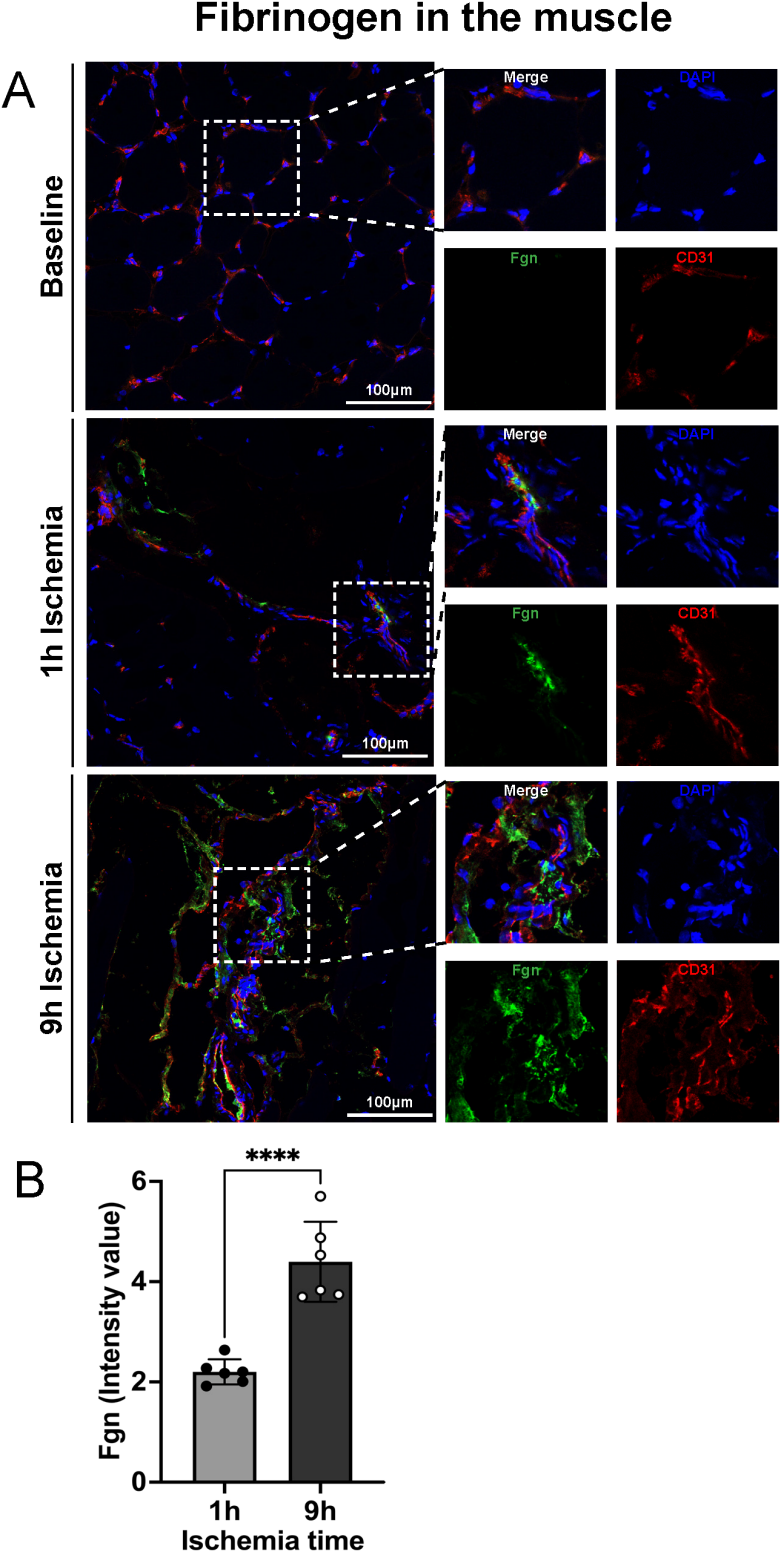
Fibrinogen extravascular deposition after prolonged ischemia followed by reperfusion. **(A)** Representative images of fibrinogen deposition in skeletal muscle, DAPI (blue), Fgn (green), CD31 (endothelial cell marker, red), scale bar = 100µm, n=6/group. **(B)** Intensity value of fibrinogen positive staining, in 3 non-overlapping fields/animal was quantified using Image J software, n=6/group, unpaired t-test ****P<0.0001. Values are shown as dots for each individual experiment with indication of mean ± SD.

To assess if limb ischemia/reperfusion leads to an increase of fibrin deposition in skeletal muscle, tissue samples were stained for fibrinogen. The results revealed extravascular fibrinogen deposition in the skeletal muscle, which was significantly more pronounced in the 9h ischemia group as compared to the 1h control (**Figures 2A, B**).

### 3.3 Prolonged ischemia followed by reperfusion leads to complement activation, neutrophil recruitment and NET formation in skeletal muscle tissue

**Figure 3:**
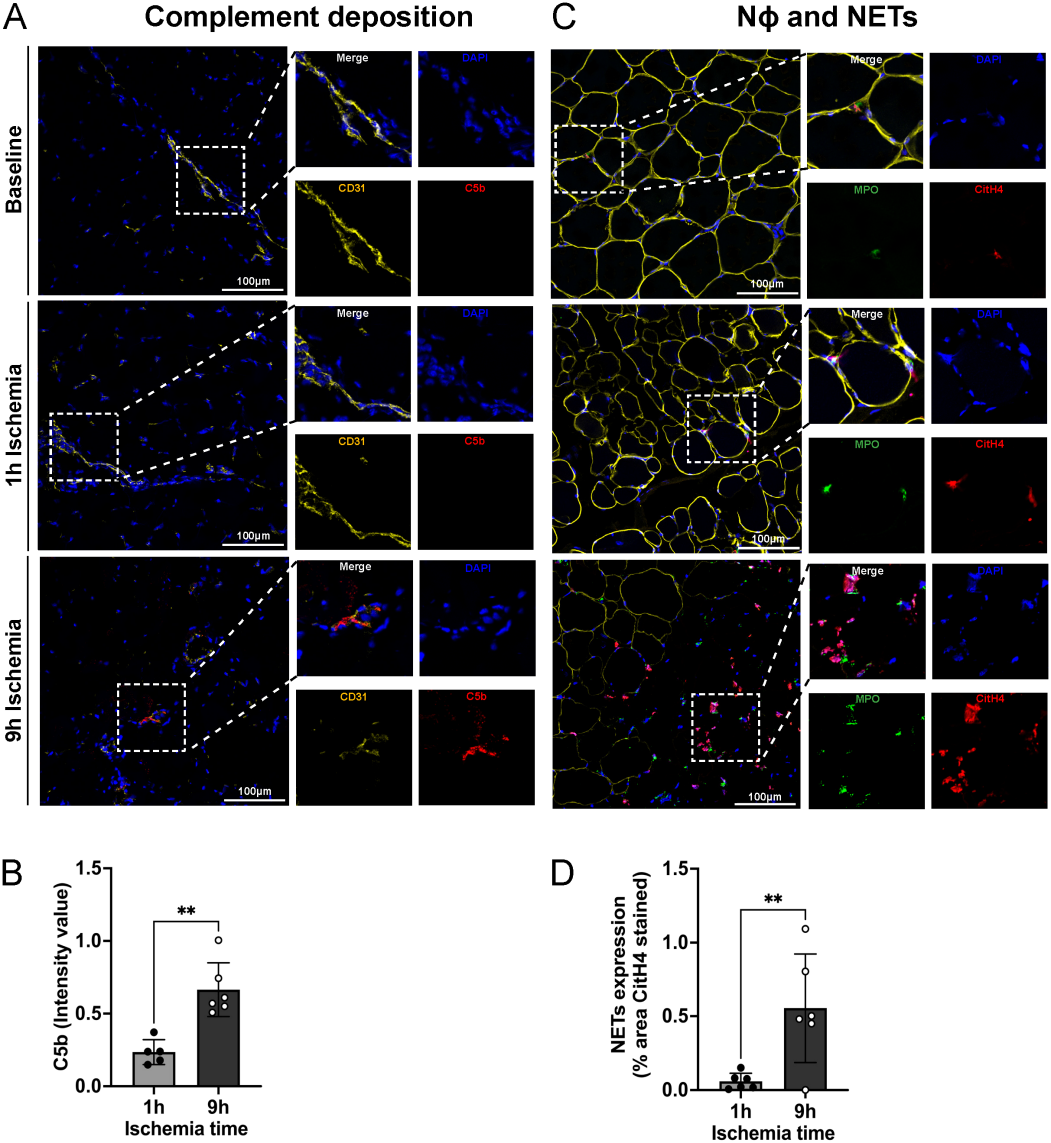
Complement deposition, neutrophils infiltration and NET-formation in skeletal muscle tissue. **(A)** Representative images of complement (C5b) deposition in skeletal muscle, DAPI (blue), CD31 (endothelial cell marker, yellow), C5b (red), Scale bar = 100µm, n=6/group. **(B)** Intensity value of C5b staining, in 3 non-overlapping fields/animal was quantified using Image J software, n=5-6/group, unpaired t-test, **P<0.01. **(C)** Representative images of neutrophil infiltration in skeletal muscle and NET formation. DAPI (blue), dystrophin (yellow), MPO (myeloperoxidase, green), CitH4 (citrullinated histone 4, red), Scale bar = 100µm, n=6/group. **(D)** NETs expression, % area CitH4 stained, in 3 non-overlapping fields/animal were quantified using Image J software, n=6/group, unpaired t-test, **P< 0.01. Values are shown as dots for each individual experiment with indication of mean ± SD.

The complement system and neutrophils exert pivotal roles in skeletal muscle tissue damage upon ischemia followed by reperfusion. To assess the interplay between complement and netting neutrophils, we performed immunofluorescence staining for the complement factor C5b and neutrophils releasing NETs. As shown in **Figures 3A, B**, 9h ischemia followed by reperfusion led to a significantly higher C5b deposition as compared to the 1h ischemia control group. Staining for neutrophil infiltration and NET formation revealed the same picture, with significantly more staining in the 9h ischemia group compared to the 1h control (**Figures 3C, D**; **Supplementary Figure S1**).

### 3.4 Prolonged ischemia followed by reperfusion leads to increased citrullinated fibrinogen levels in plasma and in skeletal muscle

**Figure 4:**
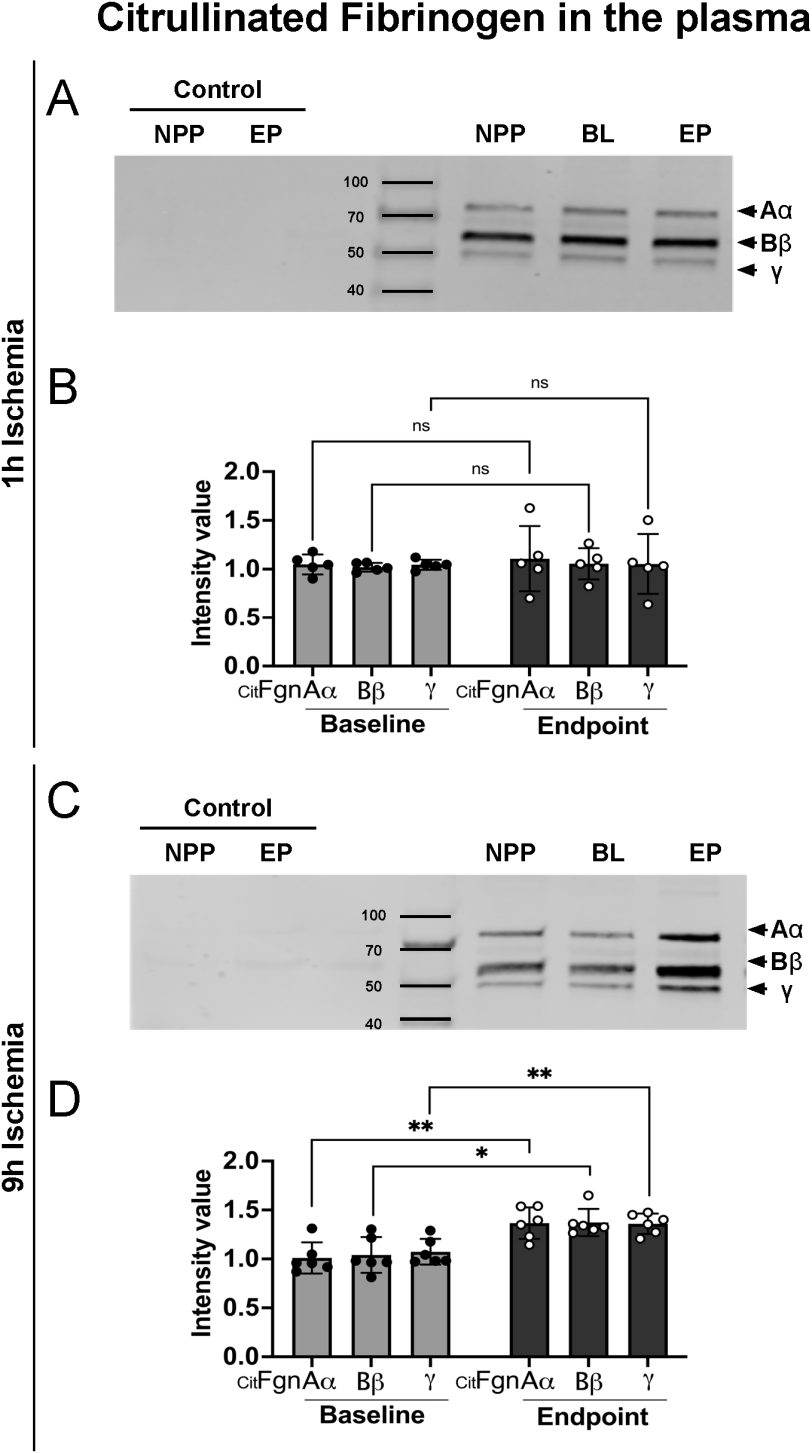
Increased citrullinated fibrinogen plasma levels after prolonged ischemia/reperfusion. **(A, C)** Representative western blots of citrullinated fibrinogen (CitFgn) from pooled normal porcine plasma (NPP) and from plasma collected at baseline (BL), and endpoint (EP) experiments in which pig limbs were subjected to 1h or 9h of ischemia, followed by 12h of *in vivo* reperfusion. Citrullinated fibrinogen was labeled with biotin-PG and immunopurified using streptavidin beads. Fibrinogen was then detected by western blot with an anti-fibrinogen antibody. As control, NPP and EP samples were immunopurified without labeling by biotin-PG. All 3 chains of fibrinogen were observed. **(B, D)** Graphs represent the relative amount of citrullinated fibrinogen for each single chain (Aα, Bβ and γ), quantified using Image J software, n=5-6/group, multiple t-tests, *P< 0.05, **P< 0.01. Values are shown as dots for each individual experiment with indication of mean ± SD.

During NET-formation, PAD is discharged into the extracellular milieu, where it can citrullinate proteins, leading to loss of protein function. To isolate citrullinated proteins from porcine plasma samples, we used an immunoprecipitation protocol based on a biotin-PG probe designed for peptidyl-citrulline recognition. Subsequently, citrullinated protein samples from both 9h and 1h ischemia groups underwent electrophoresis and were blotted for fibrinogen. The results showed a significant rise in citrullinated fibrinogen levels over baseline in the plasma collected at the end of the experiments in the 9h ischemia group, whereas this was not the case in the 1h ischemia control group (**Figures 4A–D**).

**Figure 5:**
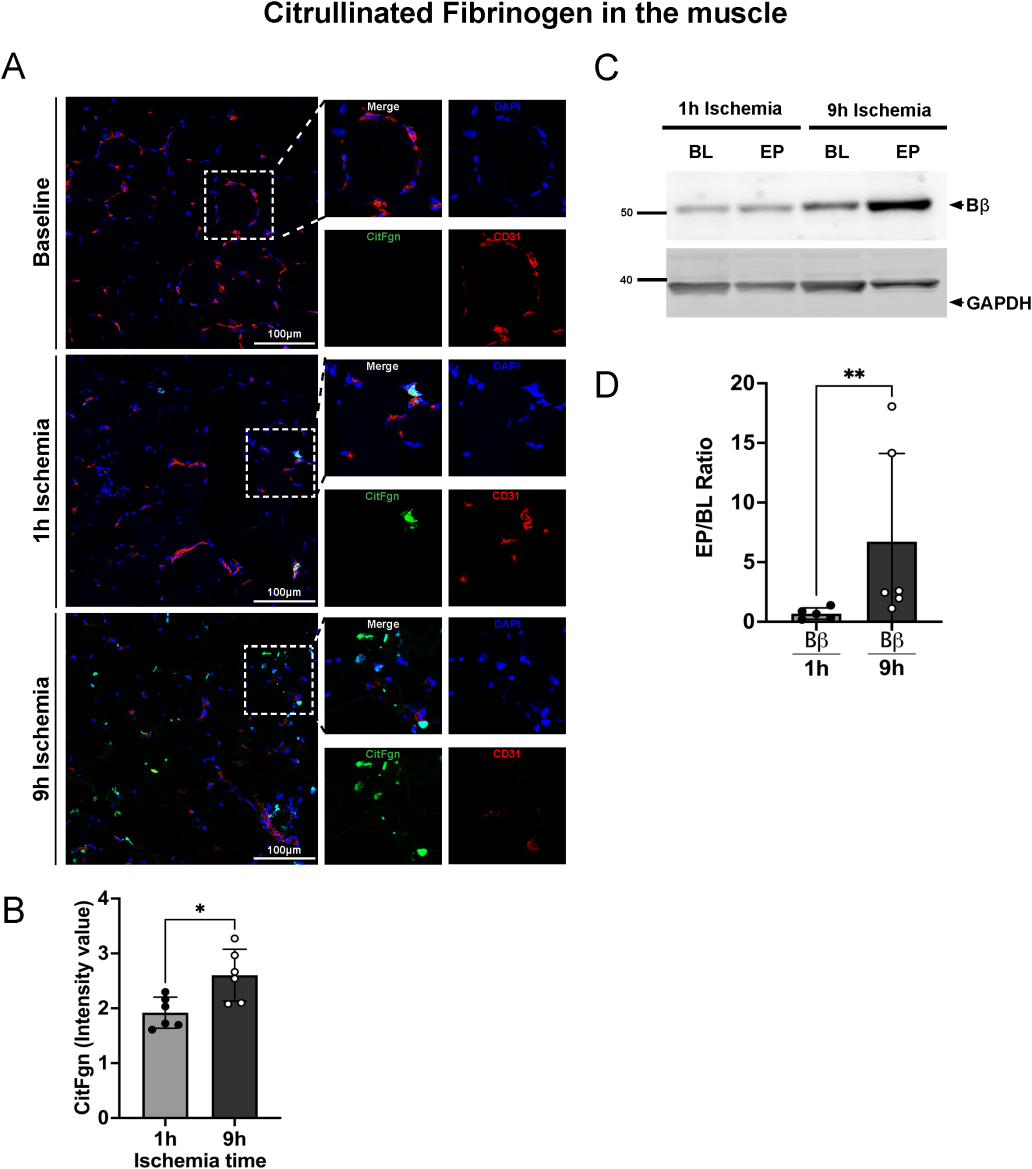
Citrullinated fibrinogen deposition in skeletal muscle tissue after prolonged ischemia/reperfusion. **(A)** Representative images of citrullinated fibrinogen deposition in skeletal muscle, DAPI (blue), CitFgn (green), CD31 (endothelial cell marker, red), scale bar = 100µm, n=6/group. **(B)** Intensity value of citrullinated fibrinogen positive staining, quantified in 3 non-overlapping fields/animal using Image J software, n=6/group, unpaired t-test *P< 0.05. **(C)** Representative western blots of citrullinated fibrinogen beta chain (Bβ CitFgn) and GAPDH from skeletal muscle tissue collected at baseline (BL), and endpoint (EP) of limbs subjected to 1h or 9h ischemia, respectively. **(D)** Graphs represent the relative amount of citrullinated fibrinogen, beta chain (Bβ chain), quantified using Image J software, n=5-6/group, unpaired t-test, Mann-Whitney test, **P< 0.01. Values are shown as dots for each individual experiment with indication of mean ± SD.

We further investigated if citrullinated fibrinogen could also be found in the tissue of limbs subjected to IRI. Skeletal muscle samples were stained for citrullinated fibrinogen. As shown in **Figure 5**, deposition of citrullinated fibrinogen was evident not only in proximity to blood vessels, but also in the parenchyma. In line with the plasma levels, a significantly higher signal for citrullinated fibrinogen was found in tissue of 9h ischemic limbs compared to 1h ischemia controls (**Figures 5A, B**). We further validated and quantified these findings using a Western blot approach. Muscle lysates from baseline, 9h and 1h experimental group underwent electrophoresis and were blotted for citrullinated fibrinogen (**Figures 5C, D**). The Western blot data confirm the analysis by immunofluorescence, showing significantly higher levels of citrullinated fibrinogen in the 9h ischemia group as compared to the 1h ischemia control. These data suggest that the inflammatory response triggered by ischemia-reperfusion not only leads to elevated citrullinated fibrinogen levels in the bloodstream, but also to its deposition in reperfused skeletal muscle.

## 4 Discussion

Despite notable progress in our understanding of the complex processes implicated in IRI and its related outcomes, significant gaps still persist (7). Here, we show ischemia-reperfusion injury triggers a dynamic response in skeletal muscle tissue, involving the complement system, NETs, and extracellular citrullination.

Our large animal model was designed to simulate a clinical situation of limb amputation and replantation after a short (1 h) or a long (9 h) period of RT ischemia of the limbs. *In vivo* reperfusion after surgical replantation was for 12 h. Based on clinical experience, this duration of reperfusion is long enough to easily detect reperfusion injury, while it is short enough to keep the pigs under constant anesthesia, so that the experiments could be performed in an acute setting, minimizing stress for the animals, which were euthanized while still under deep anesthesia.

Macroscopic analyses and clinical data, including limb weight, wet/dry ratio, and compartmental pressure, clearly showed a massive reperfusion injury in the limbs which were subjected to 9 h of ischemia before reperfusion. The observed edema formation correlated with histological muscle damage as observed microscopically by TUNEL positivity and dystrophin staining (**Figure 1**). These results in essence confirm the clinical practice, that replantation of an amputated extremity has a very low chance of success after more than 6h of ischemia (30, 31). This situation, however, is very unsatisfactory and a better understanding of the molecular mechanisms causing IRI is needed, so that drugs for its prevention or treatment can be developed.

We therefore performed an in-depth analysis of fibrin deposition, complement, NETs, and citrullination in this large animal model. First, we analyzed the local coagulation state of our animals through the analysis of fibrin deposition at baseline and after induction of IRI in the limb. Fibrinogen is mainly known as an acute phase protein, increased upon inflammation. It is one of the most important proteins involved in the coagulation cascade and prothrombotic events, but its function is not limited to coagulation (32). It is also known that fibrinogen deposition in the vascular bed or extravascular tissue plays a pivotal role in cell migration (33). Fibrinogen levels showed a significant increase in muscle tissue in the 9h ischemic group compared to the 1h ischemia control group and baseline.

The above-described results further support a role for fibrinogen beyond hemostasis. Fibrinogen is also known to facilitate cell migration through vessel walls into the tissue, with possible consequences on exacerbation of local tissue injury, including systemic procoagulant events. The latter are feared, systemic consequences of IRI, which can lead to acute respiratory distress syndrome (ARDS) and even multiorgan failure (34, 35).

Together with the increase of fibrinogen tissue deposition, activation of the complement system and in particular generation of the anaphylatoxins C3a and C5a, known as potent chemoattractants for leukocytes, further contribute to the early recruitment of neutrophils into the tissue. Complement activation represents one of the key initial events in IRI pathology and exerts a determinant role in the progression and outcome of the IRI-induced inflammation. Complement also directly damages skeletal muscle tissue by inducing cellular permeabilization and material extrusion into the extracellular milieu through the generation of C5b, which is the component initiating the membrane attack complex (MAC) (36). With our experiments we further confirm the direct action of complement in exacerbating tissue damage since we reported an increase in C5b deposition in limbs subjected to prolonged ischemia, which also showed more severe tissue damage. The complement system, once activated, works also as “alarm” system and recruits neutrophils to the inflamed sites. As deeply investigated over the years, neutrophils are the first innate immune cells to arrive in the inflamed tissue and since the last decade, they received a special attention in the study of IRI for their ability to release Extracellular Traps. NETs, discovered by Brinkmann et al. in 2004 as mechanism of the immune system to react to invading pathogens (37), are also found in the context of sterile inflammation such as IRI, where they contribute to further damaging the tissue and worsening the outcome (38). In our study we analyzed the infiltration of neutrophils and their ability to release NETs in skeletal muscle and we found a huge infiltration of netting neutrophils in the 9h ischemia group compared to the 1h control and baseline conditions. These results from our large animal model are in line with what is already known or has been proposed in literature from rodent studies (39, 40).

It is known that one of the important molecules contained in NETs are PADs enzyme, PAD2 and PAD4, which are released together with other proteins in these web-like structures. Outside the cell these enzymes are still active and can citrullinate proteins, leading to loss of their function (18). It is known from other models, that citrullination of a protein irreversibly affects its function and hypercitrullination is an abnormal citrullination process, which sparks the immune system and generates citrullinated proteins of different molecular weights.

As evidenced in several pathological models, fibrinogen is a well-known substrate for PAD2 and PAD4 and stands out as primary target for hypercitrullination. Citrullinated fibrinogen has been found increased in synovial fluids and peripheral blood of patients suffering from rheumatoid arthritis (21) and in plasma of patients affected by cancers (41), underling the strong relationship between NET formation and citrullinated extracellular proteins, Fgn included (42). To further support the close association between NETs and citrullinated fibrinogen, elevated NETs sputum levels significantly correlated with anti-citrullinated Fgn antibodies in subjects at risk for development of rheumatoid arthritis shedding a light on the possible use of citrullinated fibrinogen as diagnostic/prognostic marker (42). Given the huge amount of infiltrating netting neutrophils in our model, we decided to investigate if an increase of them could lead to an increase of citrullinated fibrinogen. Indeed, increased levels of citrullinated fibrinogen were found both in plasma and skeletal muscle tissue of limbs subjected to 9h ischemia and much less in the 1h ischemia group. Further investigations and functional analysis are needed to understand the downstream effect of increased citrullinated fibrinogen in this animal model. Preliminary data (not published) suggest that upon citrullination, fibrinogen effector functions lead to an alteration of the hemostasis. Despite it needs a deeper investigation, it looks already in line with what is known in literature, in fact it has been demonstrated *in vitro* that an increased percentage of citrullinated fibrinogen leads to a slow-down of the coagulation process (43, 44). However, once coagulation happened, the thrombi were shown to be mechanically less stable but more resistant to fibrinolysis (45, 46). These *in vitro* studies proposed a novel mechanism through which neutrophils might influence the clot lysibility upon structural alteration due to the generation of citrullinated fibrinogen via PAD enzymes. Thus, the functional alteration of a fibrin meshwork formed with higher degree of citrullinated fibrinogen is due to a composition of the fibrin network with fibers which appear to have a reduced diameter (thinner fibers), reduced bending rigidity, and porosity but an extremely increased density (43–51).

NETs are a crucial tool of the innate immune system against invading pathogens, but they also fuel proinflammatory events and cause tissue damage if activated in an uncontrolled fashion like in IRI (52). Different therapeutic approaches to inhibit NET formation and citrullination have already been investigated: Neonatal NET-inhibitory factor (nNIF) I and nNIF-related peptides inhibit key events in NET formation, including peptidyl arginine deiminase (PAD) activity, neutrophil nuclear histone citrullination, and nuclear decondensation (53); DNase 1 selectively cuts the extracellular DNA and has been approved for therapeutic intervention as Pulmozyme (Dornase alfa) by Genentech in cystic fibrosis; and the chemical Cl-amidine irreversibly inhibits PAD enzymes through covalent modification at their active sites (54). However, the therapeutic approaches developed so far focus on the total inhibition of PAD and consequently NETs. This may be a double-edged sword since PAD enzymes are also involved in various biological processes, including protective immunity, regulation of gene expression, and cell differentiation. Inhibiting all these mechanisms could impair many biological processes and cause more harm than being beneficial (55). Furthermore, PAD-KO mice are more susceptible to bacterial infections than mice which do not lack this enzyme family, suggesting that inhibition of PADs and subsequent inhibition of NETs may render patients more susceptible to infections (55, 56).

Our study opens the idea of therapeutic approaches against extracellular citrullination that specifically inhibit PAD once outside the cell to keep the physiological effector functions of intracellular PAD and NETs, fundamental in several biological process regulation and in infectious diseases while avoiding the downstream proinflammatory effects due to hypercitrullination (57). This new therapeutic avenue could be investigated in a model similar to the one we used in this study. Like this, an extension of the ischemic time before replantation might be possible and beneficial in several clinical scenarios like extended trauma surgery on extremities, myocardial infarction and allotransplantation.

## Data Availability

The raw data supporting the conclusions of this article will be made available by the authors, without undue reservation.
