# Supplementary material for: Neutrophil extracellular traps and citrullinated fibrinogen contribute to injury in a porcine model of limb ischemia and reperfusion
Source: Front Immunol. 2024 Sep 9;15:1436926. doi: 10.3389/fimmu.2024.1436926 (PMC11416929; doi:10.3389/fimmu.2024.1436926)
Supplement: Supplementary file 1 [file Image1.pdf]

frontiers

Supplementary Material

Neutrophil Extracellular Traps and citrullinated fibrinogen contribute to injury in a porcine model of limb ischemia and reperfusion

Valentina Zollet[1,2], Isabel Arenas Hoyos[1,2,3], Stefanie Hirsiger[3], Bilal Ben Brahim[1], Mariafrancesca Petrucci[1,2], Daniela Casoni[4], Junhua Wang[1], Rolf Spirig[5], Kay Nettelbeck[4], Luisana Garcia[4], Lena Fuest[3], Esther Vögelin[3], Mihai Constantinescu[3], Robert Rieben[1*]

*** Correspondence:** Prof. Robert Rieben, PhD: robert.rieben@unibe.ch

1.1 Supplementary Figures

Supplementary Figure 1: Nϕ and NETs

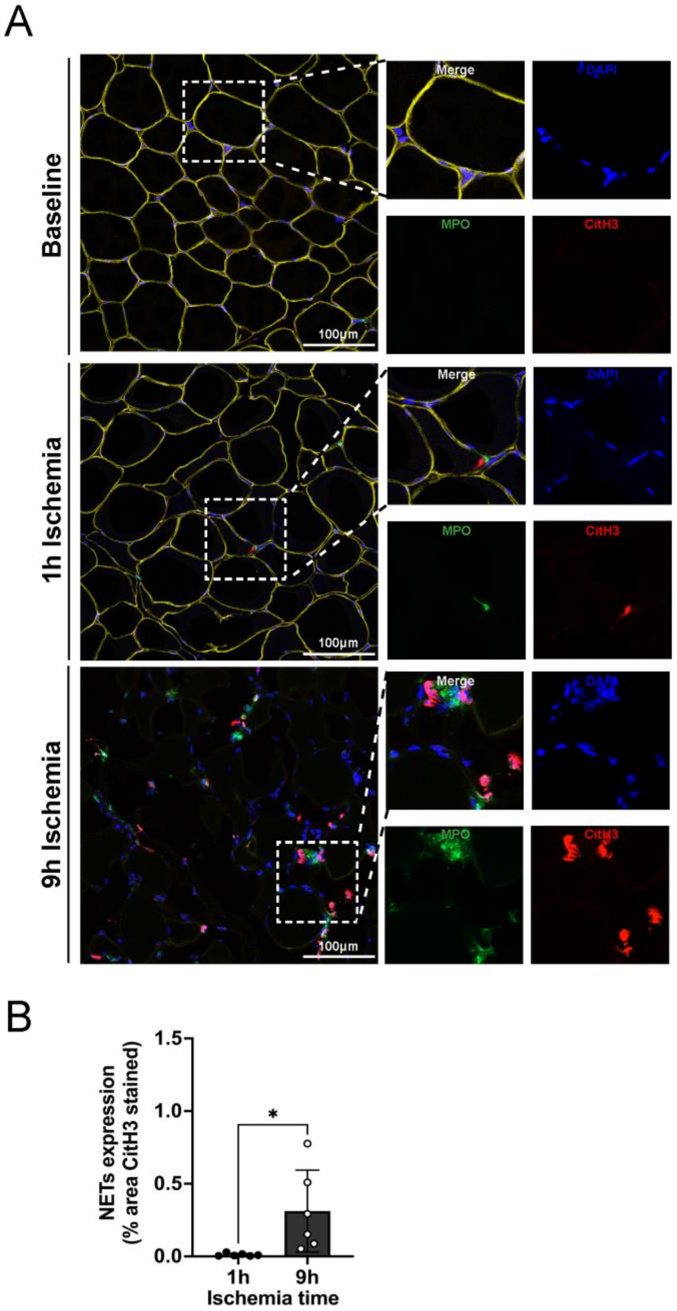

Supplementary Figure 1. Neutrophil infiltration and NET-formation in skeletal muscle tissue. (A) Representative images of neutrophil infiltration in skeletal muscle and NET formation. DAPI (blue), dystrophin (yellow), MPO (myeloperoxidase, green), CitH3 (citrullinated histone 3, red), scale bar = 100µm, n=6/group. (B) NETs expression, % area CitH3 stained, in 3 non-overlapping fields/animal were quantified using Image J software, n=6/group, unpaired t-test, *P<0.05. Values are shown as dots for each individual experiment with indication of mean ± SD.
